# Protocol for an observational study to assess the impact of pharmacogenetics on outcomes in vascular surgery (PROSPER)

**DOI:** 10.1136/bmjopen-2024-088456

**Published:** 2025-05-06

**Authors:** Kerry Anne Burke, Selman Mirza, Stuart Wright, Nicholas S Greaves, William G Newman, John H McDermott

**Affiliations:** 1 Manchester Centre for Genomic Medicine, St Mary’s Hospital, Manchester University NHS Foundation Trust, Manchester, UK; 2 Division of Evolution, Infection and Genomics, School of Biological Sciences, The University of Manchester, Manchester, UK; 3 Centre for Biostatistics, School of Health Sciences, The University of Manchester, Manchester, UK; 4 Manchester Centre for Health Economics, The University of Manchester, Manchester, UK; 5 Manchester Vascular Centre, Manchester Royal Infirmary, Manchester University NHS Foundation Trust, Manchester, UK

**Keywords:** Vascular surgery, GENETICS, VASCULAR SURGERY, Chronic Limb-Threatening Ischemia

## Abstract

**Abstract:**

**Introduction:**

Patients with chronic limb-threatening ischaemia (CLTI) are often prescribed clopidogrel in order to reduce their risk of major adverse limb and cardiovascular events. Clopidogrel is metabolised by the CYP2C19 enzyme and genetic variations in *CYP2C19* are common. These variants can influence an individual’s ability to metabolise clopidogrel to its active metabolite. Few studies have investigated the relationship between patient genotype and outcomes in vascular surgery. This work aims to establish the relationship between patient genotype and outcomes after revascularisation in patients with CLTI who are prescribed clopidogrel. It will consider whether pharmacogenetics can be used to ensure patients are prescribed effective medications to optimise their outcomes.

**Methods and analysis:**

This is an observational cohort study of patients undergoing lower limb surgical, endovascular or hybrid revascularisation for CLTI at Manchester University NHS Foundation Trust. Patients taking clopidogrel post-procedure, as well as those prescribed a non-clopidogrel based medication regimen, will be recruited prior to or shortly after revascularisation. Patients will undergo *CYP2C19* genotyping and will be followed up using online records. The study has 90% power to detect 114 amputations with a target sample size of 483 participants. The primary outcomes are risk of amputation at 1 year and a composite endpoint for the risk of major adverse limb events (MALE) or death from any cause at 1 year. Secondary outcomes are risk of MALE at 1 year, risk of major adverse cardiovascular events (MACE) or death from any cause at 1 year, death within 30 days of revascularisation, minor re-interventions at 1 year, total number of re-interventions at 1 year and rate of systemic or gastrointestinal bleed at 1 year.

Risk of amputation, MALE and MACE will be analysed using Cox models. All remaining outcomes will be analysed using negative binomial models. Potential competing events for the risk of amputation will be investigated as part of a sensitivity analysis. Patients given a non-clopidogrel-based medication will be compared as an additional analysis.

**Ethics and dissemination:**

Manchester University Research Ethics Committee approval obtained as part of the Implementing Pharmacogenetics to Improve Prescribing (IPTIP) trial process (IRAS 305751). The results of the study will be published in a peer-reviewed journal and presented at international conferences.

**Registration:**

This work is a sub-protocol for the IPTIP study which is registered as ISRCTN14050335.

**STRENGTHS AND LIMITATIONS OF THIS STUDY**

- A low attrition rate is anticipated as patient information will be extracted from e-health records, which will increase the validity of the results.
- A comparator group who are prescribed a non-clopidogrel-based medication will allow us to investigate whether these patient’s outcomes differed from those prescribed a clopidogrel-based medication during the same period.
- As this is an observational study, there is no patient randomisation and estimates of risk are likely to be confounded by important patient characteristics.

## Introduction

 Patients with chronic limb-threatening ischaemia (CLTI) have significant lower extremity arterial disease and are known to be at high risk of major adverse limb events (MALE), including loss of vessel patency, need for surgical intervention and major amputation.^1^ They are also at a significantly increased risk of major adverse cardiovascular events (MACE), including myocardial infarction, cerebrovascular events and death.^1^ Clopidogrel is an anti-platelet agent which is widely used to reduce the risk of both MALE and MACE in patients with CLTI.^2 3^ It is a thienopyridine pro-drug which is metabolised by the CYP2C19 enzyme in the liver. Genetic variations in *CYP2C19* are common and can influence an individual’s ability to metabolise clopidogrel to its active metabolite.^4^

Research studies in both cardiac and stroke medicine have demonstrated significantly worse outcomes in patients treated with clopidogrel who are poor metabolisers of CYP2C19 and major guidelines have advocated the role of genetic testing in these specialities.^5^,^7^ Research into the impact of *CYP2C19* alleles in vascular surgery is much more limited but does suggest an association between poor metabolisers of CYP2C19 and adverse outcomes in patients taking clopidogrel.^8 9^

This protocol describes an observational cohort study which aims to establish the relationship between patient genotype and outcomes after revascularisation in patients with CLTI who are prescribed clopidogrel. It will consider whether pharmacogenetics can be used to ensure patients are prescribed effective medications to optimise their outcomes.

## Methods and analysis

This is an observational cohort study involving inpatients and outpatients at Manchester University NHS Foundation Trust (MFT), to assess whether genotype is associated with clinical outcomes following revascularisation for patients with CLTI who are prescribed clopidogrel. CLTI is defined as lower extremity ischaemic rest pain and/or tissue loss present for more than 2 weeks.^10^ A comparator group who are prescribed non-clopidogrel-based medication regimens will also be recruited. A statistical analysis plan has been provided as a supplement to this article. This work is a sub-protocol for the Implementing Pharmacogenetics to Improve Prescribing trial (IPTIP) study.^11^

### Primary outcomes

1. Risk of amputation at 1 year, defined as the time to amputation from revascularisation.
2. Composite of MALE in the index limb (amputation above the ankle, major limb re-intervention (graft revision, thrombectomy or thrombolysis), loss of graft/vessel patency) or death from any cause at 1 year.

### Secondary outcomes

1. Minor re-intervention (angioplasty, stent).
2. MALE events (amputation above the ankle, major limb re-intervention (graft revision, thrombectomy or thrombolysis), loss of graft/vessel patency) at 1 year.
3. Total re-interventions.
4. Death within 30 days.
5. MACE events (myocardial infarction, cerebrovascular event or all-cause death) at 1 year.
6. Rate of systemic or gastro-intestinal bleeding.

### Inclusion criteria

- Patients awaiting revascularisation (either surgical, endovascular or hybrid) for CLTI or acute-on-chronic limb ischaemia, OR who have had a revascularisation procedure in the past 3 months.
- No previous revascularisation in the study leg 3 months prior to this intervention (excluding diagnostic angiograms).
- Patients over the age of 18 years who are able to consent for themselves.

### Exclusion criteria

- Patients receiving long-term, full-dose anticoagulation post-procedure (does not include low-dose rivaroxaban).
- Acute limb ischaemia, aneurysmal disease, vasculitis, Buerger’s disease.
- Patients are being managed conservatively without revascularisation.
- Patients who are pregnant, breastfeeding, on chemotherapy or radiotherapy.
- Patients with less than 6 months life expectancy.

Patient recruitment is expected to run from August 2023 until August 2026. Eligible participants will be provided with a patient information leaflet and will sign a consent form if they wish to enrol. A trained member of the research team will then take either a single blood sample or sputum sample, at the preference of the patient. Baseline demographic details will be collected during this time. Beyond this, participants will not be asked to do anything further for the study or to attend any future study visits. The blood or sputum sample will then be transported to the Manchester Centre for Genomic Medicine via existing and secure clinical pathways for internal specimen transport. DNA will be extracted and quantified by the North West Genomic Laboratory Hub, a NHS ISO15189 accredited laboratory. DNA samples will be labelled with the Study ID and stored at −20°C until genotyping. Genotyping will be undertaken using the AgenaTM iPLEX PGx 74 assay which can determine *CYP2C19* genotype status. All participants will have details about their past medical history, surgical history and prescribed medications collected from the Greater Manchester Care Record (GMCR). This information and the genetic data will then be linked to a secure database. The data will be anonymised so that researchers will not be able to identify individual patients.

The past medical history, surgical history and prescribed medication of participants will be recorded throughout the study period using the GMCR, NHS, MFT and other online records. The total follow-up period will be 2 years. Once the data collection period is complete, the participant genotype will then be added to the final dataset within the secure research environment, linked by the Study ID. The final anonymised dataset will include the Study ID, patient demographics, past medical and surgical history, prescription record, patient genotype and metaboliser status.

Participants can withdraw consent at any time without giving any reason, without their care or legal rights being affected, as participation in the research is voluntary. Patients who withdraw their consent to be part of the study will have their data removed from the final analysis pipeline and their DNA sample will be destroyed.

### Data statement

An anonymised version of the genetic dataset will be stored in a data repository with no limitations on access or use. Genotype data will be deposited in the Figshare repository^12^ at the end of the study following publication. Data will be open access and will be entirely anonymised, with no study ID.

### Patient and public involvement

During protocol development, the study design and methodology were discussed with the patient and public involvement and engagement with the research team, called Vocal, at MFT. This was done in a focus group session. The results of the study will be provided back to the Vocal group. Patients participating in the study are provided with an option on the consent form to receive a summary of the study results.

### Ethics and dissemination

Manchester University Research Ethics Committee approval as obtained was part of the IPTIP trial process (IRAS 305751). The results of the study will be published in a peer-reviewed journal and presented at international conferences.

## Supplementary material

10.1136/bmjopen-2024-088456 online supplemental file 1
