# Supplementary material for: Protocol for an observational study to assess the impact of pharmacogenetics on outcomes in vascular surgery (PROSPER)
Source: BMJ Open. 2025 May 6;15(5):e088456. doi: 10.1136/bmjopen-2024-088456 (PMC12056640; doi:10.1136/bmjopen-2024-088456)
Supplement: online supplemental file 1 [file bmjopen-15-5-s001.docx]

**Statistical Analysis Plan**

**Full/ Long title of the Study:** *Protocol for an Observational Study to Assess the Impact of Pharmacogenetics on Outcomes in Vascular Surgery*

**Short Study title/ Acronym:** PROSPER

**Funding is provided** by the National Institute for Health and Care Research [NIHR301748], the Genomic Medicine Service Alliance, and the NIHR Biomedical Research Centre.

**SAP version number with dates: V1.1 11.03.2025**

**Protocol version number and date: V3.3 09.03.2024**

**Roles and responsibility:**

| **Chief Investigator:** | | |
| --- | --- | --- |
| Name: Professor William Newman  Manchester Centre for Genomic Medicine  Manchester University NHS Foundation Trust |  |  |
| **Co-Investigator:** |  |  |
| Name: Dr Kerry Burke  Manchester Vascular Centre  Manchester University NHS Foundation Trust  **Co-Investigator:** |  |  |
| Name: Dr John McDermott  Manchester Centre for Genomic Medicine  Manchester University NHS Foundation Trust |  |  |
|  |  |  |
| **Co-Investigator:** |  |  |
| Name: Dr Nicholas Greaves  Manchester Vascular Centre  Manchester University NHS Foundation Trust |  |  |
|  |  |  |
| **Health Economist:** |  |  |
| Name: Dr Stuart Wright  Manchester Centre for Health Economics  University of Manchester |  |  |
|  |  |  |
| **Statistician:** |  |  |
| Name: Mr Selman Mirza  Centre for Biostatistics  University of Manchester |  |  |

# Table of contents

[Table of contents 3](#_Toc192597346)

[Abbreviations 4](#_Toc192597347)

[1 Introduction 5](#_Toc192597348)

[1.1 Background and rationale 5](#_Toc192597349)

[1.2 Objectives 5](#_Toc192597350)

[2 Methods 6](#_Toc192597351)

[2.1 Study design 6](#_Toc192597352)

[2.2 Sample size 6](#_Toc192597353)

[2.3 Timing of outcome assessments 6](#_Toc192597354)

[2.4 Timing of final analysis 6](#_Toc192597355)

[3 Statistical Principles 6](#_Toc192597356)

[3.1 Confidence intervals (CI) and level of statistical significance 6](#_Toc192597357)

[3.2 Analysis populations 7](#_Toc192597358)

[4 Study Population 7](#_Toc192597359)

[4.1 Screening 7](#_Toc192597360)

[4.2 Eligibility 7](#_Toc192597361)

[4.3 Recruitment 7](#_Toc192597362)

[4.4 Withdrawal/follow-up 8](#_Toc192597363)

[4.5 Baseline patient characteristics 8](#_Toc192597364)

[5 Analysis 8](#_Toc192597365)

[5.1 Outcome definitions 8](#_Toc192597366)

[5.1.1 Primary outcomes 8](#_Toc192597367)

[5.1.2 Secondary outcomes 9](#_Toc192597368)

[5.2 Analysis methods 9](#_Toc192597369)

[5.2.1 Primary outcome analysis 9](#_Toc192597370)

[5.2.2 Secondary outcome analysis 9](#_Toc192597371)

[5.2.3 Alternative methods 10](#_Toc192597372)

[5.2.4 Sensitivity analysis 10](#_Toc192597373)

[5.3 Missing data 10](#_Toc192597374)

[5.4 Additional analyses 11](#_Toc192597375)

[5.5 Harms 11](#_Toc192597376)

[5.6 Statistical software 11](#_Toc192597377)

[6 APPENDICES 12](#_Toc192597378)

[6.1 Appendix A: Flow diagram template 12](#_Toc192597379)

[6.2 Appendix B: Analysis groups 13](#_Toc192597380)

[7 REFERENCES 14](#_Toc192597381)

# abbreviations

CIF Cumulative Incidence Function

CLTI Chronic Limb Threatening Ischaemia

CVA CerebroVascular Accident

DNA DeoxyriboNucleic Acid

GI Gastro-Intestinal

IRR Incidence Rate Ratio

KM Kaplan-Meier

LoF Loss-of-Function

LTFU Lost-To-Follow-Up

MACE Major Adverse Cardiovascular Events

MALE Major Adverse Limb Events

MFT Manchester University NHS Foundation Trust

MI Myocardial Infarction

MVC Manchester Vascular Centre

PH Proportional Hazards

RMST Restricted Mean Survival Time

# Introduction

## Background and rationale

Patients with chronic limb threatening ischaemia (CLTI) have significant lower extremity arterial disease and are known to be at high risk of major adverse limb events (MALE), including loss of vessel patency, need for surgical intervention and major amputation^1^. They are also at a significantly increased risk of major cardiovascular events (MACE), including myocardial infarction, cerebrovascular events and death^1^. Clopidogrel is an anti-platelet agent which is widely used in order to reduce the risk of both MALE and MACE in patients with CLTI^2,3^. It is a thienopyridine pro-drug which is metabolised by the *CYP2C19* enzyme in the liver. Genetics variations in *CYP2C19* are common and can influence an individual’s ability to metabolise clopidogrel to its active metabolite^4^.

Research studies in both cardiac and stroke medicine have demonstrated significantly worse outcomes in patients treated with clopidogrel who are poor metabolisers of *CYP2C19*, and major guidelines have advocated a role of genetic testing in these specialties^5–7^. Research into the impact of *CYP2C19* alleles in vascular surgery is much more limited, but does suggest an association between poor metabolisers of *CYP2C19* and adverse outcomes in patients taking clopidogrel^8^.

PROSPER is an observational cohort study which aims to establish the relationship between patient genotype and outcomes after revascularisation in patients with CLTI who are prescribed clopidogrel. It will consider whether pharmacogenetics can be used to ensure patients are prescribed effective medications to optimise their outcomes.

## Objectives

The primary objective is to assess whether genotype is associated with clinical outcome following vascular intervention for individuals prescribed clopidogrel.

The secondary objective is to establish a genotyped cohort of patients presenting with PAD undergoing endovascular or surgical intervention.

The additional objective is to assess whether clinical outcome following vascular intervention for individuals prescribed clopidogrel differs to individuals prescribed other medication and how this interacts with each genotyped cohort.

# Methods

## Study design

The study will be an observational cohort study which includes patients recruited both retrospectively and prospectively. The cohort is split into four groups based on genotype and drug treatment (see table 1 appendix B).

## Sample size

The desired sample size has been calculated assuming an average amputation rate of 0.35 at 1 year^11^, a hazard ratio for *CYP2C19* loss of function (LoF) allele carriers for the primary endpoint of 2.0 and prevalence of LoF allele carriers of 26.2%^12,13^. Based on the Schoenfeld method for proportional hazards^14^ (PH), the power calculation using a significance level of 5% and 90% statistical power, 114 events would be needed to identify an effect. Taking into consideration the average amputation rate, the study would need to recruit 326 participants receiving a clopidogrel-based medication regimen. Assuming 25% of all admissions receive a non-clopidogrel based antiplatelet therapy with 10% missing data, 483 participants would need to be recruited to the study in total. Sample size calculations were performed in R software (version 4.4.2).

## Timing of outcome assessments

For time-to-event outcomes, events will be recorded when they occur throughout the study period. All remaining outcomes will be assessed at 1 year from baseline.

## Timing of final analysis

Analyses will take place after the 1-year follow-up period has been completed. All outcomes will be analysed collectively at the time of final data *identification and extraction* which will be September 2027, with all information entered into the database and the full database is cleaned and locked.

# Statistical Principles

## Confidence intervals (CI) and level of statistical significance

Analyses will be conducted using two-sided 95% confidence intervals at the 5% significance level. No formal adjustments will be made for multiplicity since each primary outcome uses a different hypothesis.

## Analysis populations

The analysis population for each outcome will be the complete-case population (i.e. excluding any patients for whom data are or become unavailable for research).

The data analyses will be based on anonymised patient-level data. Patients who wish to withdraw their consent to allow their data to be used will not be included in the analyses.

# Study Population

## Screening

Eligible participants will be identified by the reviewing surgeon and will be approached by their direct clinical or research team (CRN nursing support and sub-investigators), the study will be discussed, and a patient information leaflet (PIL) will be provided. Thereafter, if the patients wish to enrol, a consent form will be signed, blood samples for DNA will be taken and the initial recruitment proforma will be completed.

## Eligibility

Inclusion criteria:

1. Patients awaiting revascularisation (either surgical, endovascular or hybrid) for CLTI or acute-on-chronic limb ischaemia, OR who have had a revascularisation procedure in the past 3 months.
2. No previous revascularisation in the study leg 3 months prior to this intervention (excluding diagnostic angiograms).
3. Patients over the age of 18 years who are able to consent for themselves.

Exclusion criteria:

1. Patients receiving long term, full-dose anticoagulation post-procedure (does not include low-dose rivaroxaban).
2. Acute limb ischaemia, aneurysmal disease, vasculitis, Buerger's disease.
3. Patients being managed conservatively without revascularisation.
4. Patients who are pregnant, breastfeeding, on chemotherapy or radiotherapy.
5. Patients with less than six months life expectancy.

## Recruitment

We plan to recruit participants from the Manchester Vascular Centre (MVC) at the Manchester Foundation Trust (MFT), a specialist unit which covers a population of 2.6million and performs over 600 interventions each year.

A flow diagram showing the number of participants screened, eligible, declined to participate, and withdrawn will be used to summarise the recruitment of participants.

## Withdrawal/follow-up

Patients can withdraw consent for their data to be used in the analyses. In these cases, patients will be summarised and excluded from the analyses.

Patients that withdraw consent will be censored from the date of withdrawal. Patients that are lost-to-follow-up (LTFU) will be censored from the date of last known contact.

## Baseline patient characteristics

We will use the participants date of revascularisation as the baseline time of entry to the study. Comparison of baseline characteristics, treatment group, time to amputation and follow-up time by carrier status for all participants. Numerical measures will be summarised by the mean, standard deviation (continuous and symmetrical), or the median and interquartile range (ordinal or not symmetrical). Categorical variables will be summarised by frequencies and percentages. Characteristics between carrier status groups will be compared using t-tests or chi-square tests as appropriate. Baseline characteristics will include:

- Age (years)
- Gender
- Ethnicity
- Diabetes mellitus
- Renal disease
- Smoking status
- Limb Rutherford classification
- Prior intervention to the index leg

# Analysis

## Outcome definitions

### Primary outcomes

1. Risk of amputation at 1-year. Time to amputation will be measured in months from the date of revascularisation. Subjects will be followed until the primary endpoint, with censoring at time of death from any cause, withdrawal, LTFU or at 1-year.
2. Composite of Major Adverse Limb Events (MALE) in the index limb [amputation above the ankle, major limb re-intervention (graft revision, thrombectomy or thrombolysis), loss of graft/vessel patency] or death from any cause at 1-year. Subjects will be followed until the composite endpoint, with censoring at time of withdrawal, LTFU or at 1-year.

### Secondary outcomes

1. MALE in the index limb at 1-year. Subjects will be followed until the composite endpoint, with censoring at time of death from any cause, withdrawal or LTFU at 1-year.
2. MACE events at 1-year (MI, CVA or all-cause death). Subjects will be followed until the outcome endpoint, with censoring at time of withdrawal, LTFU or at 1-year.
3. Death within 30 days from revascularisation.
4. Minor re-interventions (angioplasty, stent) at 1-year.
5. Total number of re-interventions at 1-year.
6. Rate of systemic or gastro-intestinal (GI) bleed at 1-year.

## Analysis methods

All primary and secondary outcomes will be compared by carrier status for the clopidogrel group (i.e. A vs B, see table 1 appendix B). The number of primary and secondary events by carrier status will be reported with crude incidence rates of events (with 95% CIs) at 1-year. The Cumulative Incidence Function (CIF) will be used to estimate incidence rates to account for potential competing risks^15^.

### Primary outcome analysis

1. Risk of amputation at 1-year will be estimated using Cox regression to compare carrier status for the clopidogrel group.

The primary analysis will be adjusted for age, gender, diabetes status and Rutherford classification for severity. The hazard ratio (HR) with corresponding 95% confidence intervals (CIs) and p-values will be reported.

The assumption of proportional hazards (PH) will be assessed by examining the Schoenfeld residuals.

1. Composite of MALE in the index limb or death from any cause at 1-year will be investigated using the same approach as risk of amputations.

### Secondary outcome analysis

- 1. MALE events will be analysed in the same manner as the corresponding primary outcome but death from any cause will be censored.
  2. MACE events at 1-year will be analysed in the same manner as the primary outcome for risk of amputations.
  3. Death within 30 days from revascularisation. Frequency with percentages will be presented by carrier status. No formal comparison between carrier status will be made.
  4. Minor re-interventions at 1-year will be analysed using a zero-inflated negative binomial model to compare carrier status. The IRR with corresponding 95% CIs and p-values will be reported.
  5. Total re-interventions at 1-year will use the same approach as minor re-interventions.
  6. Rate of systemic or GI bleed at 1-year will use the same approach as minor re-interventions.

### Alternative methods

If the PH assumption cannot be satisfied, the primary outcome at 1-year will be analysed using Restricted Mean Survival Time (RMST)^16^. Both Cox regression and RMST can produce estimates of the HR in the presence of competing risks.

For secondary outcomes measured as count data, if the ‘alpha’ parameter and likelihood ratio test from the negative binomial model suggests no issue with overdispersion, then all relevant secondary outcomes at 1-year will be examined using a zero-inflated Poisson model.

### Sensitivity analysis

Potential competing events are defined as death from any cause and a sensitivity analysis will be included for the risk of amputation at 1-year. CIF and 1-KM function plots for the primary outcome will be compared to investigate potential competing risks. If this comparison suggests the potential for competing risks, then the sensitivity analysis will examine the sub-distribution hazard model^15^. We will report the HR with the corresponding 95% CIs and p-values.

Baseline characteristics that do not form part of the primary analysis will be included in the sensitivity analysis and estimates of risk will be compared to the primary analysis.

## Missing data

The frequency and percentage of missing primary outcome data will be examined by carrier status. If the level of missing data is much higher than expected, the nature of the missing data mechanism will be investigated. We anticipate the level of missing data in our study to be low. Missing data that could be considered at least ‘missing at random’ would be few and have no significant impact on the analysis. We expect the majority of our missing data to be ‘not missing at random’ because some patients may have to discontinue treatment with clopidogrel which implies multiple imputation methods would not be suitable.

## Additional analyses

There will be a supporting analysis to examine the non-clopidogrel group to compare carrier status for the risk of amputation (i.e. C vs D, see table 1 appendix B). This treatment group will be observed during the same time period and is not expected to differ in terms of carrier status. This will provide a more robust interpretation of our results from the primary analysis. The model chosen will depend on the PH assumption and if competing risks are present for the risk of amputation at 1-year.

If our analysis for the risk of amputation shows the clopidogrel group differs in terms of carrier status, an exploratory analysis will be included to compare clopidogrel and non-clopidogrel based medications. The model chosen will depend on the PH assumption and if competing risks are present for the risk of amputation at 1-year and will make two comparisons. The first comparison will examine the clopidogrel group vs. the non-clopidogrel group for those who carry LoF alleles (i.e. A vs C, see table 1 appendix B). The second comparison will examine the clopidogrel group vs the non-clopidogrel group for non-carriers (i.e. B vs D, see table 1 appendix B).

## Harms

As this is an observational study, the risk to participants and researchers is not above that of normal clinical practice. All patient DNA samples will be stored securely within an NHS facility where the risk of DNA contamination or loss is very low.

## Statistical software

Statistical analyses will be performed using Stata or other similar software.

# APPENDICES

## Appendix A: Flow diagram template

Screened n = xxx

Consented n =xxx

Genotype testing n = xxx

Loss-of-Function alleles n = xx

No loss-of-function alleles n = xx

Ineligible n = xxx

Consented but did not participate= xx

Eligible: not consented = xx

12m events X(Y%); no event X(Y)%

Withdrawn = X(Y%); Lost F-UP = X(Y%)

12m events X(Y%); no event X(Y)%

Withdrawn = X(Y%); Lost F-UP =X(Y%)

Figure 1: PROSPER diagram – recruitment and follow-up of patients**.**

## Appendix B: Analysis groups

|  | *CYP2C19* LoF allele carrier (26.2%) | *CYP2C19* LoF allele non-carrier (73.8%) |
| --- | --- | --- |
| 1. Clopidogrel containing anti-platelet therapy (75%) | **A** | **B** |
| 1. Non-clopidogrel containing anti-platelet therapy (25%) | **C** | **D** |

Table 1: Analysis Groups within the PROSPER Study. Estimated percentages of carrier status in each group are based on historical data.

## 7 REFERENCES

1. Aboyans V, Ricco JB, Bartelink MLEL, Björck M, Brodmann M, Cohnert T, et al. 2017 ESC Guidelines on the Diagnosis and Treatment of Peripheral Arterial Diseases, in collaboration with the European Society for Vascular Surgery (ESVS): Document covering atherosclerotic disease of extracranial carotid and vertebral, mesenteric, renal, upper and lower extremity arteriesEndorsed by: the European Stroke Organization (ESO)The Task Force for the Diagnosis and Treatment of Peripheral Arterial Diseases of the European Society of Cardiology (ESC) and of the European Society for Vascular Surgery (ESVS). European Heart Journal. 2018 Mar 1;39(9):763–816.

2. Antiplatelet treatment. National Institute for Health and Care Excellence. 2023 Sept. [Internet]. [cited 2023 Oct 24]. Available from: https://cks.nice.org.uk/topics/antiplatelet-treatment/

3. Twine CP, Kakkos SK, Aboyans V, Baumgartner I, Behrendt CA, Bellmunt-Montoya S, et al. Editor’s Choice – European Society for Vascular Surgery (ESVS) 2023 Clinical Practice Guidelines on Antithrombotic Therapy for Vascular Diseases. European Journal of Vascular and Endovascular Surgery. 2023 May;65(5):627–89.

4. McDermott JH, Sharma V, Keen J, Newman WG, Pirmohamed M. The Implementation of Pharmacogenetics in the United Kingdom. In: Cascorbi I, Schwab M, editors. Precision Medicine [Internet]. Cham: Springer International Publishing; 2023. p. 3–32. Available from: https://doi.org/10.1007/164_2023_658

5. Annotation of DPWG Guideline for clopidogrel and CYP2C19 [Internet]. PharmGKB. [cited 2023 Nov 5]. Available from: https://www.pharmgkb.org/guidelineAnnotation/PA166104956

6. Abdullah-Koolmees H, van Keulen AM, Nijenhuis M, Deneer VHM. Pharmacogenetics Guidelines: Overview and Comparison of the DPWG, CPIC, CPNDS, and RNPGx Guidelines. Front Pharmacol. 2021 Jan 25;11:595219.

7. Testing could help prevent further strokes in people with gene variant. National Insititue of Health and Care Excellence. [Internet]. NICE. NICE; 2023 [cited 2023 Nov 5]. Available from: https://www.nice.org.uk/news/article/testing-could-help-prevent-further-strokes-in-people-with-gene-variant

8. Burke KA, McDermott JH, Wright SJ, Newman WG, Greaves NS. A review of clopidogrel resistance in lower extremity arterial disease. JVS-Vascular Insights. 2024 Jan 1;2:100112.

9. Wübbeke LF, Naves CCLM, Daemen JWHC, Jacobs MJ, Mees BME. Editor’s Choice – Mortality and Major Amputation after Revascularisation in Octogenarians Versus Non Octogenarians with Chronic Limb Threatening Ischaemia: A Systematic Review and Meta-Analysis. European Journal of Vascular and Endovascular Surgery. 2020 Aug;60(2):231–41.

10. Perlander A, Jivegård L, Nordanstig J, Svensson M, Österberg K. Amputation-free survival, limb symptom alleviation, and reintervention rates after open and endovascular revascularization of femoropopliteal lesions in patients with chronic limb threatening ischemia. Journal of Vascular Surgery. 2020 Dec 1;72(6):1987–95.

11. Tornio A, Flynn R, Morant S, Velten E, Palmer CNA, MacDonald TM, et al. Investigating Real-World Clopidogrel Pharmacogenetics in Stroke Using a Bioresource Linked to Electronic Medical Records. Clin Pharmacol Ther. 2018 Feb;103(2):281–6.

12. Bath PM, Woodhouse LJ, Appleton JP, Beridze M, Christensen H, Dineen RA, et al. Antiplatelet therapy with aspirin, clopidogrel, and dipyridamole versus clopidogrel alone or aspirin and dipyridamole in patients with acute cerebral ischaemia (TARDIS): a randomised, open-label, phase 3 superiority trial. The Lancet. 2018 Mar 3;391(10123):850–9.

13. Schoenfeld DA. Sample size formula for the proportional-hazards regression model. Biometrics. 1983 Jun; 39: 499-503.

14. Introduction to the Analysis of Survival Data in the Presence of Competing Risks | Circulation [Internet]. [cited 2024 Apr 10]. Available from: https://www.ahajournals.org/doi/10.1161/CIRCULATIONAHA.115.017719

15. Royston P, Parmar MK. Restricted mean survival time: an alternative to the hazard ratio for the design and analysis of randomized trials with a time-to-event outcome. BMC Medical Research Methodology. 2013 Dec 7;13(1):152.
