# Supplementary material for: Protocol for an observational study to assess the impact of pharmacogenetics on outcomes in vascular surgery (PROSPER)
Source: BMJ Open. 2025 May 6;15(5):e088456. doi: 10.1136/bmjopen-2024-088456 (PMC12056640; doi:10.1136/bmjopen-2024-088456)
Supplement: Supplementary file 2 [file bmjopen-2024-088456.reviewer_comments.pdf]

PEER REVIEW HISTORY

BMJ Open publishes all reviews undertaken for accepted manuscripts. Reviewers are asked to complete a checklist review form and are provided with free text boxes to elaborate on their assessment. These free text comments are reproduced below.

ARTICLE DETAILS

Title (Provisional)

Protocol for an Observational Study to Assess the Impact of Pharmacogenetics on Outcomes in Vascular Surgery (PROSPER)

Authors

Burke, Kerry Anne; Mirza, Selman; Wright, Stuart; Greaves, Nicholas S; Newman, William G; McDermott, John H

VERSION 1 - REVIEW

**Reviewer**	**1**
**Name**	**Tan, Xin**
**Affiliation**	**Capital Medical University Affiliated Anzhen Hospital**
**Date**	**22-Jul-2024**
**COI**	**I have no competing interests**

---

1. In the abstract, the innovation of the article needs to be highlighted, since currently CYP2C19 is a commonly used genetic testing item to determine whether patients taking clopidogrel can achieve effective results.

2. In statistical analysis, I did not see any relevant charts of hazard ratio (HR), incidence rate ratio (IRR) and Kaplan-Meier (KM) plots.

3. The limitations, such as the article used a prospective observational cross-sectional study instead of a prospective cohort study, did not discussed

4. There are too few references in the article.

**Reviewer**	**2**
**Name**	**Zhong, Yongliang**
**Affiliation**	**Capital Medical University Affiliated Anzhen Hospital**
**Date**	**25-Jul-2024**
**COI**	**no**

This paper is well-written, and I have no further comments.

**Reviewer**	**3**
**Name**	**Powell, Janet**
**Affiliation**	**Imperial College, Vascular Surgery**
**Date**	**07-Oct-2024**
**COI**	**None**

If this is a prospective study how can you include patients who have had a limb revascularization within the past 3 months? Some of the outcomes cannot be evaluated, since you cannot include patients who have already died.

Planned recruitment period not provided. What about start of recruitment and how many centres will contribute? What is the annual casel0ad at St Mary's/Manchester.

The amputation free survival rate at 1 year of 35% seems low and is not referenced. The patient cohort is not limited to CLTI patients and no definition of CLTI is provided.

ESVS guidelines do not recommend dual antiplatelet therapy and recommend clopidogrel as first choice. What proportion of your patients have other therapies? Will there be sufficient for the 4 group analysis?.

The primary outcome will be influenced by age, sex, diabetes and severity of disease and therefore it is desirable that Cox models adjusting for these factors are included in the analysis plan.

**Reviewer**	**4**
**Name**	**Le Meur, Nolwenn**
**Affiliation**	**Ecole des Hautes Etudes en Sante Publique**
**Date**	**25-Oct-2024**
**COI**	**None to declare**

The study protocol is really interesting and well written. Although some precisions would clarify the method.

P6: The 5th secondary outcomes is "MACE events at any time" but could the authors be more specific in that paragraph? Is it at 1 year (censoring at 12 monts p9 - analysis p10) or 2 years as mentioned p7 for the follow-up period?

P7 : For the past medical history, surgical history and prescribed medication of participants that will be recorded throughout the study period, do the authors have a limit back in time?

P7: The authors state that "this data will be pseudo-anonymised". What method is envisioned? Foin or double-foin algorithms?

VERSION 1 - AUTHOR RESPONSE

Reviewer: 1
Dr. Xin Tan, Capital Medical University Affiliated Anzhen Hospital
Comments to the Author:
1. In the abstract, the innovation of the article needs to be highlighted, since currently CYP2C19 is a commonly used genetic testing item to determine whether patients taking clopidogrel can achieve effective results.
An additional sentence has been added to the abstract to highlight this.

2. In statistical analysis, I did not see any relevant charts of hazard ratio (HR), incidence rate ratio (IRR) and Kaplan-Meier (KM) plots.
As the study is not completed, we would not include these charts at this stage. The planned use of HRs and KM plots is mentioned as a part of the analyses. We have added a further comment in the secondary outcomes (minor re-interventions) to make this clearer (see tracked changes).

3. The limitations, such as the article used a prospective observational cross-sectional study instead of a prospective cohort study, did not discussed.
The text has been changed to clarify that this is a cohort study.

4. There are too few references in the article.
References have been added while addressing the other reviewer comments.

Reviewer: 2
Dr. Yongliang Zhong, Capital Medical University Affiliated Anzhen Hospital
Comments to the Author:
This paper is well-written, and I have no further comments.

Reviewer: 3
Prof. Janet Powell, Imperial College
Comments to the Author:
If this is a prospective study how can you include patients who have had a limb revascularization within the past 3 months? Some of the outcomes cannot be evaluated, since you cannot include patients who have already died.
This is considered in our statistical analysis. Part 1 of the primary analysis will censor those who died, and we will then we check for competing risks. Part 2 will include those who died from any cause and compare results from parts 1 and 2 to see if these deaths had an impact on our results.
This work is intended as a pilot study to inform a larger-scale randomised control trial. The ability to include patients with recent revascularisation is necessary in order to achieve the desired recruitment numbers with the resources available.

Planned recruitment period not provided. What about start of recruitment and how many centres will contribute? What is the annual casel0ad at St Mary's/Manchester.
The planned recruitment period has been added to the protocol. This is believed to be feasible as the Manchester Vascular Centre covers a population of 2.6million and performs over 600 interventions each year.

The amputation free survival rate at 1 year of 35% seems low and is not referenced.
0.35 is the expected event rate (as you would see with an amputation free survival rate of 65%) and is believed to be in keeping with the literature. Two references have been added.

The patient cohort is not limited to CLTI patients and no definition of CLTI is provided.
The cohort only includes CLTI patients, a definition has been added.

ESVS guidelines do not recommend dual antiplatelet therapy and recommend clopidogrel as first choice.
The ESVS guidelines state that it is reasonable to recommend a short course of DAPT following endovascular revascularisation.

What proportion of your patients have other therapies? Will there be sufficient for the 4 group analysis?
This is addressed in the sample size calculation and table 1 (i.e. including 25% non-clopidogrel). We are not comparing 4 groups, rather 2 groups based on their carrier status while adjusting for 2 different treatment options (i.e. clopidogrel and non-clopidogrel).

The primary outcome will be influenced by age, sex, diabetes and severity of disease and therefore it is desirable that Cox models adjusting for these factors are included in the analysis plan.
Age, sex, and diabetes are mentioned in the statistical plan on pg. 9. Rutherford classification is used to account for severity.

Reviewer: 4
Dr. Nolwenn Le Meur, Ecole des Hautes Etudes en Sante Publique
Comments to the Author:
The study protocol is really interesting and well written. Although some precisions would clarify the method.

P6: The 5th secondary outcomes is "MACE events at any time" but could the authors be more specific in that paragraph? Is it at 1 year (censoring at 12 monts p9 - analysis p10) or 2 years as mentioned p7 for the follow-up period?
It is at one year, this has been clarified in the text.

P7 : For the past medical history, surgical history and prescribed medication of participants that will be recorded throughout the study period, do the authors have a limit back in time?

As follow-up is done with electronic records, it will include all available information with no time limit.

P7: The authors state that "this data will be pseudo-anonymised". What method is envisioned? Foin or double-foin algorithms?
The text has been amended to clarify this to anonymised data.

VERSION 2 - REVIEW

**Reviewer**	**1**
**Name**	**Tan, Xin**
**Affiliation**	**Capital Medical University Affiliated Anzhen Hospital**
**Date**	**03-Feb-2025**
**COI**	

The paper has been well revised, and I have no other comments.

**Reviewer**	**3**
**Name**	**Powell, Janet**
**Affiliation**	**Imperial College, Vascular Surgery**
**Date**	**18-Jan-2025**
**COI**	

1 Since some of the responses depend on the statistical analysis plan, this should be published as a supplement to the paper.

2 The retrospective inclusion of patients has been partially justified but means that it would be better to remove prospective from the title and replace it with pilot.

VERSION 2 - AUTHOR RESPONSE

Reviewer: 1
Dr. Xin Tan, Capital Medical University Affiliated Anzhen Hospital

Comments to the Author:
The paper has been well revised, and I have no other comments.

Reviewer: 3
Prof. Janet Powell, Imperial College

Comments to the Author:

1 Since some of the responses depend on the statistical analysis plan, this should be published as a supplement to the paper.

A statistical analysis plan has been added as a supplement as suggested.

2 The retrospective inclusion of patients has been partially justified but means that it would be better to remove prospective from the title and replace it with pilot.

The authors agree with the reviewer's comments that this is not a pilot study. The vast majority of the data collection is prospective as explained in the methods.

Reviewer: 1
Competing interests: Not applicable

Reviewer: 3
Competing interests: No competing interests
